# The Effect of Death Anxiety on Work Passion: Moderating Roles of Work Centrality and Work Connection

**DOI:** 10.1177/00302228241236227

**Published:** 2024-02-28

**Authors:** Saeed A. AL-Dossary, Cátia Sousa, Gabriela Gonçalves

**Affiliations:** 1 Psychology Department, College of Education, 48138 University of Ha’il, Hail, Saudi Arabia; 2 School of Management, 70985 Tourism and Hospitality, University of Algarve, Faro, Portugal; 3 Centre for Research in Psychology (CIP/UAL), University of Algarve, Faro, Portugal; 4 Faculty of Human and Social Sciences, University of Algarve, Faro, Portugal

**Keywords:** death anxiety, work passion, work-family centrality, work connection, structural equation modelling, multi-group moderation analysis

## Abstract

Fear of death is an emotional manifestation of the instinct for self-preservation. Any threat to our existence induces an anxiety response. Death anxiety can trigger obsessive-compulsive behaviours, such as an obsessive passion for work. Using a sample of 314 participants (68.2% female), with a mean age of 38.97 years (SD = 10.36), this study sought to observe the predictive effect of death anxiety on work passion, as well as the moderating effect of work-family centrality and connection on the relationship between anxiety and passion. The results revealed that death anxiety negatively affects harmonious passion, and positively affects obsessive passion. Work centrality did not moderate the influence of death anxiety on harmonious and obsessive passion. Nonetheless, work connection moderated the influence of death anxiety on harmonious passion. The negative influence of death anxiety on harmonious passion was greater in a group with high work connection than a group with low connection.

## Introduction

‘The Greeks didn’t write obituaries. They only asked after a man died: “Did he have passion?”’

– Serendipity (2001)

Confronting death and the anxiety generated by the knowledge of its inevitability is a universal psychological dilemma for humans (Lehto & Stein, 2009). Therefore, human beings must learn to live and adapt to the awareness of their own finitude (Becker, 1973). Death is a complex physio-psychosocial event that can induce anxiety due to the conflict between the desire for self-preservation and the awareness of the inevitability of death. The theory of terror management postulates that the awareness of the inevitability of death originates a potentially overwhelming terror, and this terror must be managed through the development of anxiety buffer strategies, such as, for example, investing in self-esteem to generate the feeling that we have value in the world (Greenberg et al., 1986; Hui et al., 2014), the desire to leave a legacy, and to be remembered after death (Waggoner et al., 2023). In this sense – and given that society has increasingly attributed importance to the meaning of work rather than mere survival, as work is one of the most important spheres of life, and that it occupies a large part of the time and energy of individuals (e.g. Chen et al., 2020) – we believe that work passion can be enhanced by death anxiety. However, when excessive, this anxiety can lead to negative consequences such as aggressiveness (e.g., Chaiwutikornwanich, 2023) and/or obsessive-compulsive behaviours (Becker et al., 2023; Li et al., 2018). For example, death anxiety can stimulate obsessive work attitudes and behaviours, namely an obsessive passion for work. The dualistic model of passion states that individuals can develop an obsessive passion for an activity they enjoy. Thus, it is expected that death anxiety exerts a positive influence on obsessive passion.

Although the literature on the antecedents of work passion is extensive (Smith et al., 2023), investigations that assess the predictive role of death anxiety in this construct are scarce or non-existent. The research described here integrates the literature on the existential threat and the impact on attitudes towards work, namely on work passion, and thus contributes to the understanding of individual differences and the psychological experiences of workers as variables of the processes that occur at work and in organisations. Thus, the objective of this study is to observe the predictive effect of death anxiety on work passion, as well as the mediating effect of centrality and connection to work, in the relationship between these variables.

## Death Anxiety

‘Death anxiety’ encompasses the adverse and menacing emotions experienced when contemplating death and dying, characterized by feelings of fear, unease, worry, and apprehension associated with mortality (e.g., Upenieks, 2023). The literature is not unanimous in its definition, and on the contrary presents different views (Lehto & Stein, 2009). Death anxiety refers to an individual's emotional response when experiencing negative thoughts related to death (Waite et al., 2022). For Dadfar and Lester (2017) death anxiety is a fear of the death of oneself. Abdel-Khalek and Tomás-Sábado (2005) frame death anxiety as the result of awareness about death. Death anxiety is thus presented as a complex construct that includes fear of death of oneself, fear of death of others, fear of the dying process, fear of the dead, fear of the treatment of one's body after death, and fear of possibilities of life after death (Menzies & Menzies, 2023). Cai and colleagues (2017), however, state that fear and anxiety are distinct theoretical constructs. Although fear and anxiety share emotional and behavioural consequences, fear emphasises negative emotional reactions to visible, specific events or actual threats, while anxiety emphasises negative reactions to nonspecific, potential, and distant threats (Vermetten et al., 2002). Yalom (1980) emphasised that fear of death was the basis of death anxiety; that everyone would feel fear of death, but only some would feel anxiety. Some individuals have greater anxiety problems because their anxiety buffers have never developed or have developed suboptimally (Yetzer & Pyszczynski, 2019). From the perspective of Terror Management Theory (TMT: Greenberg et al., 1986) ineffective anxiety buffers, caused by genetic predispositions, insecure attachment, life stressors, self-esteem, meaning creation and formation or maintenance of an incoherent identity, and instability in childhood and adolescence may hinder the development of an effective system of protection against anxiety (Yetzer & Pyszczynski, 2019) resulting in psychological vulnerability (Maxfield et al., 2014).

For Li et al. (2018) death anxiety is not a sudden emotion, but a continuous state throughout life, and the degree of anxiety related to death varies from person to person (Bengtson et al., 1977). A low degree of anxiety does not affect the lives of individuals, but when it becomes excessive it can cause damage to physical and mental health. For example, death anxiety plays a crucial role in obsessive-compulsive disorders such as OCD (Menzies et al., 2019), depressive disorders (e.g., Menzies et al., 2019), eating disorders (e.g., Goldenberg et al., 2005; Le Marne & Harris, 2016), and psychotic disorders (e.g., Easden et al., 2022) among others.

## Dualistic Model of Work Passion

Contemporary perspectives on work passion highlight its numerous positive outcomes, such as perceived meaning, persistence, overall success, enthusiasm, financial gain, and happiness (Pollack et al., 2020; Vallerand & Houlfort, 2003). Pollack et al. (2020) meta-analysis identifies three research avenues on work passion: general passion (Baum & Locke, 2004), role-based passion (Cardon et al., 2009), and the dualistic model of passion (Vallerand et al., 2003). These approaches offer distinct definitions of passion: (1) General passion: Focuses on work-related positive feelings, asserting that love for work motivates perseverance and maintenance of these feelings (Baum & Locke, 2004); (2) Role-Based Passion: Involves consciously accessible positive feelings associated with entrepreneurial activities, activating self-regulation processes to achieve goals (Cardon et al., 2009; Thorgen & Wincent, 2013, 2015); (3) Dualistic Model of Passion: Defines passion as a strong inclination towards an enjoyable activity, integrating an affective component and internalization within a person’s identity (Vallerand & Houlfort, 2003).

This model introduces components beyond positive emotions, requiring an activity, like work, to be an integral part of one’s identity to become an object of passion. Passion can take two forms based on internalization: harmonious passion (intrinsically motivated and balanced) and obsessive passion (controlled and potentially conflicting with other life areas) (Vallerand et al., 2003, 2014). Harmonious passion is a voluntary, committed pursuit, while obsessive passion can be compelling and may conflict with other life aspects (Vallerand et al., 2010; Vallerand & Miquelon, 2007).

While passion can enhance motivation and wellbeing, it can also trigger negative emotions and obsessive behaviors, potentially disrupting a balanced life (Orgambídez-Ramos et al., 2014). Perspectives differ based on how an activity is internalized, leading to distinct antecedents and consequences (Chen et al., 2020; Curran et al., 2015; Pollack et al., 2020). However, both perspectives signify a strong commitment to the activity (Salessi et al., 2017).

## Work-Family Centrality and Work Connection

Work-family centrality is linked to an individual's judgment about the importance they assign to each role they play, which varies among individuals. Some prioritize roles that enhance self-esteem and well-being (Carr et al., 2008; Xie et al., 2017). Those with high work-family centrality invest more time and energy into work than their family life, while those with low work-family centrality prioritize family-related tasks (Xie et al., 2017).

For the MOW group (1987), work is seen as a complex psychological construction shaped by personal experiences and choices. Work centrality refers to an individual's belief in the value of work, observed either absolutely or relatively (MOW, 1987). Absolute work centrality relates to work's significance in shaping an individual’s self-image, affecting their time commitment and emotional involvement in work-related activities (Coda & Fonseca, 2004; MOW, 1987).bib_mirt_1987

## Death Anxiety, Work Passion, and Work-Family Centrality

The fear of death often drives individuals to engage in various activities that help them avoid confronting this reality. Several theories, including reprioritization theory, symbolic immortality, gerotranscendence theory, and terror management theory, explore this aspect. For instance, reprioritization theory suggests that negative life events lead individuals to reassess their goals, seeking a reorganization of life priorities (Heckhausen, 1997). Symbolic immortality refers to the human desire for continuity despite the certainty of death (Lifton, 1974, 1979). This pursuit gives meaning to our existence, connecting us with others both in life and after death (Lifton & Mitchell, 1996). The theory of gerotranscendence posits that aging prompts a shift in perspective from materialism to a more transcendent view, fostering a quest for higher consciousness (Tornstam, 1999).

Terror management theory (TMT) asserts that awareness of death induces overwhelming terror, disrupting daily functioning (Pyszczynski et al., 2004). Individuals manage this terror by investing in self-esteem and cultural beliefs to affirm their value (Greenberg et al., 1986; Kosloff et al., 2019). TMT suggests that in modern societies, where success and pleasure are paramount, reminders of mortality prompt people to tightly grasp their material goals to maintain self-worth (Sheldon & Kasser, 2008).

Studies propose various buffers against death anxiety, such as attachment styles, intimate relationships, and even consumerism (Gasiorowska et al., 2018; Menzies & Menzies, 2023). Thoughts of mortality often increase materialistic aspirations and consumption desires, serving as a tranquilizer in modern societies (Arndt et al., 2004). This intensifies the pursuit of wealth, fame, and image (Kosloff & Greenberg, 2009). Money, according to Zaleskiewicz et al. (2013), acts as a buffer against death anxiety, resulting in increased desire for money. The work, for example, allows for social connection, a sense of belonging, creativity, respect, economic and social status, among other emotional and psychological benefits (e.g., Combs et al., 2023), making it a powerful tool in responding to the need for symbolic immortality and coping with death anxiety (Waggoner et al., 2023). As individuals remain professionally active, they maintain their self-efficacy, not identifying with the aging stage and not perceiving their abilities as declining.

However, these anxiety buffers might lead to long-term obsessive behaviors (e.g., compulsive shopping, psychological disorders), and therefore, the individual may develop an obsessive passion for work.

Obsessive passion for work, driven by internalizing work into one’s identity and seeking social acceptance and self-esteem, aligns with feelings postulated by TMT to alleviate death anxiety (Pyszczynski et al., 2004). Consequently, death anxiety is expected to positively predict obsessive work passion, especially when work is highly central to an individual’s identity. Therefore, the following research hypotheses were formulated:

H1 Death anxiety will have a negative influence on harmonious work passion.

H2 Death anxiety will have a positive influence on obsessive work passion.

H3 Work-family centrality moderates the relationship between death anxiety and harmonious work passion.

H4 Work-family centrality moderates the relationship between death anxiety and obsessive work passion.

H5 Connection to work moderates the relationship between death anxiety and harmonious work passion.

H6 Connection to work moderates the relationship between death anxiety and obsessive work passion.

**Figure 1.**
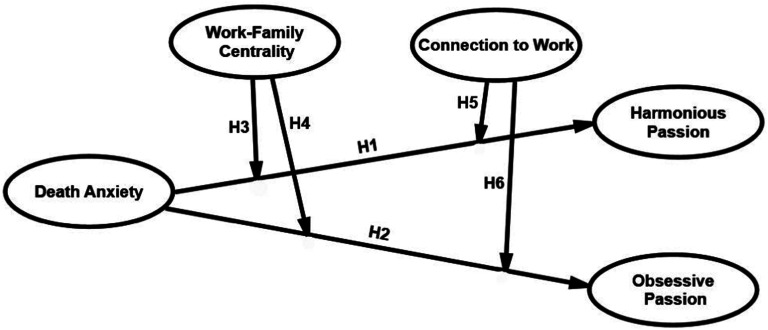
The hypothesised model.

Figure 1 illustrates the hypothesised model.

## Method

### Participants and Procedure

This study used a convenience sample using the snowball strategy. The inclusion criteria for participation were individuals over 18 years old, professionally active, and willing to participate voluntarily. Data were collected between May and July 2022. The study was approved by the Scientific Committee of Faculdade de Ciências Humanas e Sociais, Universidade do Algarve (Portugal) and assurance of ethical criteria being followed was given (e.g., information about the voluntary and anonymous nature of the study). The participants were asked to answer a self-report questionnaire with an average completion time of 12 min. Before enrolling on the study, participants received no details regarding its exact topic. The invitation did not mention that the study referred to religiosity, in order to avoid self-selection bias for religiously oriented participants. Participants responded to the online questionnaire with guaranteed anonymity. Informed consent was presented to participants at the beginning of the survey. No compensation was offered to participants and the study subject was blinded. Only the questionnaires completed correctly were considered. Data collection resulted in a sample composed of 314 people of both genders (31.8% male and 68.2% female), with an average age of 38.97 years (SD = 10.36, missing = 12). The sample includes worker participants (77.7%) and student-workers (70%). With regard to marital status, most are married/common law marriage (50%) and the rest are single (29.5%), divorced and widowed (10.5%) and the remaining participants did not indicate. Most participants do not have children (43.9%) or only have one (27.1%). The number of children ranged from zero to 8 (Mean = .91). The characterization of the sample regarding literary qualifications includes participants with basic education (3.5%), secondary education (29.9%), graduation degree (48.1%), master’s degree (14.3%) and doctorate (3.8%). The contractual relationship of most workers is effective contract (69.4%) followed by fixed-term contract (17.2%), the remaining participants are independent workers (5.7%), other situations (5.7%) or missing (1.9%). Most work full-time (92.4%) and are spread across a wide range of professional activity (e.g., military professions, administrative, operators of installations and machines). The professional activity with the highest percentage is intermediate level technicians and professionals (38.5%). Most participants reported not having had anyone close to them who had recently died (66.6%).

### Measures

#### Demographic Characteristics

To characterize the sample, the questionnaire included a set of sociodemographic questions, such as age, sex, marital status, number of children, educational qualifications, contractual relationship, type of work schedule and type of professional activity. Given the death anxiety variable, the form included the question ‘Has someone very close to you died recently?’ which showed no effect on the results.

#### Scale of Death Anxiety

The version adapted for the Portuguese population by Gonçalves et al. (2023), from the scale originally constructed by Cai et al. (2017) which was used. It is an instrument composed of 17 items that intend to assess the perception and feelings of individuals when thinking about their own death. It has a 4-dimensional scale (Dysphoria, Death Intrusion, Fear of Death, Avoidance of Death) and all items (e.g., ‘In the past month, I have often avoided thoughts or topics related to death’, ‘In the past month, whenever thinking of death, I have often gotten upset’), are operationalised on an increasing Likert-type scale of 7 values (1 - ‘I totally disagree’ to 7 - ‘I totally agree’). Cronbach’s alpha for the original scale was .86. The Portuguese adaptation present an alpha value of .89. In the current study, the alpha of the scale was .93.

#### Work Passion

Originally developed by Vallerand and Houlfort (2003), for this study, we used the adaptation of the Passion Scale for the Portuguese population (Gonçalves et al., 2014). The scale reflects the dual perspective of passion for work, being composed of two subscales of 7 items: harmonious passion (e.g. item 3: ‘This activity allows me to live memorable experiences’; item 5: ‘This activity is in harmony with the other activities in my life’) and obsessive passion (e.g., item 8: ‘I cannot live without this activity’; item 13: ‘I have almost an obsessive feeling for this activity’). Both dimensions of passion for work are measured according to a 7-point Likert scale (1 - Strongly Disagree to 7 - Strongly Agree). Scores of reliability on the originally Passion Scale have ranged from. .78 (harmonious passion) to .88 (obsessive passion). The Portuguese adaptation present an alpha value of .92 for harmonious passion and .93, for obsessive passion (Gonçalves et al., 2014). In the present study the alphas varied from .92 for harmonious passion and .90 for obsessive passion.

#### Work-Family Centrality

The work-family centrality was assessed using Carr´s et al. (2008) Work-Family Centrality Scale. In the present study, the version adapted for the Portuguese population by Marta (2018) was used. It is a scale composed of 5 items that assess the relative importance of work versus family in an individual’s life (e.g., item 1 “In my opinion, an individual’s personal life goals should be more work-oriented than family-oriented,” item 5 “Overall, I consider work to be much more important to life than family”). Responses are rated on a 7-point Likert scale (1 – strongly disagree to 7 – strongly agree), where higher means correspond to greater work centrality, and lower means correspond to greater family centrality. Cronbach’s alpha for the original scale was .93, for the Portuguese adaptation was .86, and in the present study was .80.

#### Connection to Work

To measure the connection with work, a single-item pictorial was used to assess the cognitive component of being connected with work, based on the instrument by Schultz (2001) and supported by the Inclusion of Other in the Self Scale by Aron et al. (1991) to measure connection with nature), which has been shown to have good validity (Zickfeld & Schubert, 2016). Participants were asked to indicate from 1 to 7, how close they felt to work, by selecting the diagram that best described their relationship with work.

Alpha reliability coefficients for all constructs were greater than the recommended value of .70, ranging from 0.80 for the work centrality scale to 0.93 for the death anxiety scale (Nunnaly & Bernstein, 1994) (Table 1).

**Table 1.** Results of Measurement Model.

Construct	Items	Standardised Factor Loading	t-Value	Cronbach’s Alpha
Death anxiety	DA1	.790	fixed	0.925
	DA2	deleted	—	
	DA3	.749	13.404	
	DA4	.749	6.379	
	DA5	.802	14.519	
	DA6	.893	fixed	
	DA7	.611	10.606	
	DA8	.682	12.031	
	DA9	.731	fixed	
	DA10	.774	13.555	
	DA11	.719	fixed	
	DA12	.730	12.737	
	DA13	.833	14.050	
	DA14	.848	14.965	
	DA15	.941	16.695	
	DA16	.893	15.027	
	DA17	.805	13.569	
Harmonious passion	HP1	.709	fixed	0.919
	HP2	.810	13.252	
	HP3	.790	16.283	
	HP4	.784	12.829	
	HP5	.700	11.463	
	HP6	.873	14.201	
	HP7	.784	12.840	
Obsessive passion	OP1	.728	fixed	0.901
	OP2	.747	13.925	
	PO3	.761	15.215	
	PO4	.869	14.416	
	OP5	.839	13.928	
	OP6	.771	12.753	
	OP7	.527	8.734	
Work-family centrality	WC1	.772	fixed	0.797
	WC2	.859	8.799	
	WC3	deleted		

All loadings are significant at *p* 0.001.

#### Data Analysis

Data were analysed using SPSS version 25 and AMOS version 23. Before testing the study hypotheses, the data was checked for accuracy, missing values, outliers, and normality assumption. The study hypotheses were verified using a two-step approach. First, the overall measurement model was examined using confirmatory factor analysis to assess the reliability and validity of the constructs. Second, the structural model of the effect of death anxiety on both types of passion at work was then examined using full structural equation modelling. To test the moderating effects of work centrality and connection to work, multi-group analysis was conducted.

## Results

### Preliminary Analyses

In preparation of the data for analysis, they were screened for accuracy, missing values, outliers, and normality assumption. There were some missing values and two cases had missing values of more than 10%. Little’s test indicated that the data were missing completely at random (χ^2^ = 152.65, df = 276, *p* = 1). Therefore, the two cases were removed from the analysis and mean imputation was used to replace the missing data.

Multivariate outliers were identified by computing each case’s Mahalanobis distance; a case is considered to be a multivariate outlier if the probability associated with its D^2^ is 0.001 or less (Tabachnick & Fidell, 2001). Eleven cases were identified as multivariate outliers and thus deleted from the analysis. After deleting these cases, the remaining data contained 301 cases. Normality testing is based on the value of skewness and kurtosis (Tabachnick & Fidell, 2001). The values ranged between −0.168 to 1.40 for skewness, and between −0.343 and 1.74 for kurtosis. Hence, the normality distribution was acceptable.

### Measurement Model

The measurement model was tested using CFA with maximum likelihood estimation to assess the validity and reliability for each item. Model fit was evaluated with the following indices: Comparative Fit Index (CFI), Standardized Root Mean Square Residual (SRMR), and Root Mean Square Error of Approximation (RMSEA) with a 90% confidence interval (90% CI). To determine the acceptable model fit of these indices, the cut-off values are: CFI ≥.90, and SRMR, RMSEA ≤ .08 (Byrne, 2016; Hu & Bentler, 1999). The model did obtain a good fit. The results indicated that item 2 in the death anxiety scale and item 3 in the work centrality were found to have very poor reliabilities. Therefore, they were deleted. In addition, the results indicated the model fit could be improved by allowing the correlation error between item 1 and 2, and between item 3 and 4 in the harmonious passion scale, and between item 8 and 9, item 8 and 10, item 12 and 13, and item 13 and 14 in the obsessive passion. The modified model provided good fit (CFI = 0.914, SRMR = 0.0548, RMSEA = 0.065 [90%CI = 0.060–0.70]). The results of factor loadings are shown in Table 2. All factor loadings were statistically significant and ranged from .53 to .94, indicating that each item was well represented by the factors. Descriptive statistics and correlation between the constructs are shown in Table 2.

**Table 2.** Correlation Analysis and Descriptive Statistics.

Constructs	1	2	3	4	5	Mean (SD)
1. Death anxiety	1					1.92 (1.05)
2. Harmonious passion	−.099	1				5.19 (1.24)
3. Obsessive passion	.139*	.487**	1			3.16 (1.40)
4. Work-family centrality	.038	.347**	.439**	1		3.30 (1.45)
5. Work connection	−.007	.092	.265**	.397**	1	3.90 (1.79)

**p-value < .01; * *p*-value < .05.

### Structural Model

**Figure 2.**
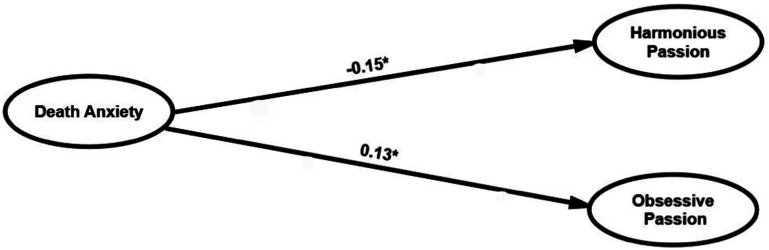
Structural model results. * *p*-value < .05.

The effect of death anxiety on both types of work passion were analysed using structural equation modelling. The model provided good fit to the data (GFI = 0.924, CFI = 0.968, SRMR = 0.062, RMSEA = 0.054 [90% CI = 0.043–0.064]). Figure 2 shows the results of the structural model with the standardised path coefficients. The results revealed that death anxiety negatively affects harmonious passion (ß = - 0.15, *p* < .05), and positively affects obsessive passion (ß = 0.13, *p* < .05).

### Moderating Effect of Work Connection and Work-Family Centrality

Multigroup analysis was employed to investigate the moderating effect of work-family centrality and work connection on the relationship among the variables in the proposed model. We performed analysis by differentiating the subjects into a group with high work centrality and work connection, and another one with low work-family centrality and work connection based on their average values. Table 3 represents the moderating effect of work-family centrality and work connection on the relationship between death anxiety and work passion.

**Table 3.** Moderation Effect of Work Centrality and Work Connection.

	High Work Centrality Group *n* = 155		Low Work Centrality Group *n* = 146		z-Score
	Estimate	*p*	Estimate	*p*	
H3	−0.090	0.123	−0.197	0.031	0.983
H4	0.141	0.097	0.131	0.160	0.084

	High Connection to Work Group *n* = 177		Low Connection to Work Group *n* = 124		z-Score
	Estimate	*p*	Estimate	*p*	
H5	−0.227	0.003	−0.042	0.608	−1.672*
H6	0.092	0.313	0.193	0.062	−0.730

*p*-value < .10.

The moderation analysis revealed that work-family centrality did not have a significant moderating effect on the influence of death anxiety on harmonious and obsessive work passion. Nonetheless, work connection has a significant moderating effect on the influence of death anxiety on harmonious work passion. The negative influence of death anxiety on harmonious passion was significantly greater in a group with a high work connection than a group with a low work connection. On the other hand, no significant moderating effect of work connection was found on the relationship between death anxiety and obsessive passion.

## Discussion

Studies on death anxiety have proliferated in the literature in recent years (e.g., Chen et al., 2020; Damirchi et al., 2020; Guner et al., 2021; Kuo et al., 2022). However, despite the importance that work represents in people's lives, few studies have addressed the effect of death anxiety on attitudes towards work, namely, work passion. Thus, it was the objective of this study to investigate the effect of death anxiety on work passion. The study also examined the moderating roles pf work centrality and connection to work in this relationship. According to the proposed investigation model, it was possible to confirm hypotheses 1, 2 and 5, but not hypotheses 3, 4 and 6. Death anxiety had a predictive power on work passion. On the one hand, it had a negative influence on harmonious passion (H1), and on the other, it was a positive influence on obsessive passion (H2). As proposed and expected, the obsessive passion for work results from a fixation on the work activity and its internalisation in the individual’s identity, which provides feelings of social acceptance and self-esteem. These feelings are, according to the TMT, important to reduce death anxiety by operating as mitigating factors for the dread caused by the inevitable realisation that we all die (Pyszczynski et al., 2004). Based on the TMT, anxiety must be managed through buffering strategies aimed at increasing self-esteem which consequently allows the individual to feel that they have value, or that they leave something in this world after departing. Several theories point to the possibility of leaving a legacy, either through new generations, or music, art, and literature (e.g., Waggoner et al., 2023). Others exhibit some buffer strategies to face anxiety, such as consumerism, perfectionism, and the accumulation of goods, among other things (e.g., Arndt et al., 2004). However, regardless of these strategies, some individuals may have higher levels of anxiety that can lead to negative consequences, namely the development of obsessive behaviours. In this study it was possible to observe that, in fact, death anxiety is a significant predictor of obsessive passion, thus addressing the literature that refers to the development of obsessive-compulsive behaviours because of excessive anxiety (e.g., Menzies et al., 2019). This result is reinforced by the negative relationship between death anxiety and harmonious passion. This investigation also expected to observe the moderating role of variables related to work, such as work centrality and work connection. Only H5 was confirmed, which indicates that the connection with work is a negative moderator of the relationship between death anxiety and harmonious passion. A greater connection or centrality to work could operate as an inducer of obsessive passion in people with higher levels of anxiety. In this case, negative moderation indicates that more harmonious behaviours are not triggered by anxious people or people with greater connection and/or centrality to work. This may mean that individuals who do not avoid the thought of death (i.e., think of it as something natural and inevitable, without fear of death) develop a psychological balance in their existence, and are better able to make their lives authentic and meaningful (Wong, 2008; Yalom, 2008), by valuing work and family and consequently developing harmony with work. While no one can avoid death, focussing on certain activities or behaviours can temporarily alleviate anxiety and provide a sense of control. However, individuals can also develop maladaptive behaviours (e.g., avoidance behaviours, seeking safety) (Menzies & Veale, 2022) or self-compensating behaviours, such as working excessively. Although the literature on the consequences of obsessive passion is unclear (with studies showing a variety of negative, positive and null outcomes (e.g., Astakhova & Porter, 2015; Curran et al., 2015; Vallerand et al., 2007, 2008)), individuals obsessively passionate about work are haunted by a partial and incomplete integration of the Self, and thus seek to pursue their passion as a way to satisfy in part the external contingencies of the Self (e.g., status, respect, need for approval [see Crocker & Wolfe, 2001]). Consequently, obsessively passionate people structure their lives and activities by placing too much importance on their passion (i.e., their work) to sustain their sense of self, and they position their passion in structural opposition to other life activities (Bélanger et al., 2013). This situation leads to the continuous loss of resources by individuals (e.g., Houlfort et al., 2013; Stenseng et al., 2011), but much of that resource allocation tends to be ‘wasted’ on anxiety and rumination about one’s passion (e.g., Donahue et al., 2012; Forest et al., 2011). Furthermore, while people are generally able to recover the daily loss of resources (Binnewies et al., 2009), obsessively passionate individuals have greater difficulty recovering resources due to their intense focus on themselves. Obsessively passionate people exhibit a closed mind toward resource-generating opportunities and activities that might otherwise prevent emotional burnout (Amarnani et al., 2019).

## Practical Implications

Although some studies indicate that obsessively passionate workers perform better (e.g., Schellenberg et al., 2022), the consequences of obsessive behaviours can affect the physical and psychological wellbeing of individuals. Thus, it is important for organisations to recognize the price of obsessive passion. The absence of an awareness and of dysfunctional forms of passion may result in a positive view of obsessive passion, with it being thought of as a high-performance enhancer. It is important to equip managers to identify obsessive behaviours, in this case, obsessive passion, as well as to monitor presenteeism, overtime, use of email outside working hours, and so on (Amarnani et al., 2019).

Confrontation with the inevitability of death and existentialist concerns are inherent to the human condition and easily activated (Becker, 1973; McCabe & Erdem, 2021). In recent decades, interest in understanding the impact of this dimension of life, as well as other associated aspects, such as religiosity, spirituality, religious values, and mourning, on human behaviour, particularly in the work context, has increased. (e.g., Benefiel et al., 2014; David & Iliescu, 2022; Pawar, 2016).

Studies have shown that instead of promoting an organisational culture of embarrassment and ‘denial of death’, taking death for granted and the expression of workers’ religious and existentialist beliefs can contribute to reducing internal conflict and anxiety (e.g., Kubler-Ross, 1969; Testoni et al., 2020) and increase the meaning of life and promote people’s self-esteem (e.g., Shakil & Yousaf, 2015). For example, HR policies for grieving and integrating religion into the workspace (e.g., flexible hours to accommodate the need to meditate, pray, attend religious services, etc.) can positively affect behaviours at work (e.g., Ahmad et al., 2019; David & Iliescu, 2022; Houghton et al., 2016).

## Limitations and Future Studies

Although this investigation benefits from the current literature, there are still some limitations. First, the search method was relatively simple. To further explore the causal relationship between these variables, future studies may apply the longitudinal study method and/or manipulate death anxiety (e.g., increasing mortality salience) in the laboratory. For example, classic short-answer open-ended questions or death-related image and audio materials can be used to launch the relevance of mortality (Burke et al., 2010). In addition to obsessive passion, other organisational variables can be deepened, such as burnout or workaholism. Second, this study adopted a self-report method to measure death anxiety; responses may have been susceptible to social desirability effects (e.g., when respondents reported less death anxiety than they actually felt). Third, human reactions to death anxiety are dynamic and multifaceted (e.g., Kesebir, 2014; Zhang et al., 2019), thus additional moderating factors may influence the relationship between death anxiety and obsessive work passion.

We consider that the justification presented for the unconfirmed hypotheses is legitimate and supported by the literature, but we believe that other studies should facilitate better clarification of the moderating effect of these variables. These include attributes such as neuroticism and variables such as spirituality, religiosity, and beliefs, as well as attitudes and values towards work. For example, the Protestant work ethic that establishes an ideological commitment to hard work (even if it does not have a Protestant base) (e.g., van Hoorn & Maseland, 2013; Momeyer, 1988; Plusnin et al., 2018; Solomon et al., 1991) can help to explain in a more sustained way the difference between effect of work centrality and obsessive and harmonious passion. The individual’s degree of commitment to work guides his/her life, i.e., he/she may prioritize work without, however, being a buffer for anxiety or an effect of anxiety in the face of death. In other words, you can have a passionate connection, but not an obsessive one.

## Conclusion

By accepting our mortality, we affirm our intent to invest our energy and time in living a good life rather than defending ourselves against inevitable death. Ideally, accepting death should free us from anxiety, and give us energy to live with enthusiasm and purpose. Thus accepting death is one of the pillars of a good life (Wong, 2008). However, death anxiety is always present as the spectre of death looms over us, reminding us of our mortality. Although some individuals manage to overcome this feeling, others tend to develop maladaptive behaviours. It is fundamental that human beings learn to live with this inevitability, and that organisations begin to integrate in their HR policies, a space to meet the needs of employees in the field of mortality (e.g., Jacobson & Beehr, 2022).
